# Sociodemographic, Mental, and Lifestyle Correlates of Mediterranean Diet Adherence in Children Aged 6–9 Years: Findings from a Large National Survey in Greece

**DOI:** 10.3390/epidemiologia6030032

**Published:** 2025-07-03

**Authors:** Georgia-Eirini Deligiannidou, Konstantinos Papadimitriou, Aikaterini Louka, Sousana K. Papadopoulou, Maria Mentzelou, Maria G. Grammatikopoulou, Evmorfia Psara, Christos Kontogiorgis, Olga Alexatou, Constantinos Giaginis

**Affiliations:** 1 Department of Nutritional Sciences and Dietetics, School of Health Sciences, International Hellenic University, 57001 Thessaloniki, Greece; edeligia@med.duth.gr (G.-E.D.); kpapadimitriou@ihu.gr (K.P.); souzpapa@gmail.com (S.K.P.);; 2 Department of Medicine, School of Health Sciences, Democritus University of Thrace, 68100 Alexandroupolis, Greece; ckontogi@med.duth.gr; 3 Department of Food Science and Nutrition, School of Environment, University of Aegean, 81100 Myrina, Greece; loukathy612@gmail.com (A.L.); maria.mentzelou@hotmail.com (M.M.); fnsd21013@fns.aegean.gr (E.P.); fnsd23003@fns.aegean.gr (O.A.)

**Keywords:** child nutrition, maternal influences, Mediterranean diet, depression, anxiety, mental health, cross-sectional study, Greece

## Abstract

**Background/Objectives**: The Mediterranean diet (MD) is well-studied for its health-promoting effects, while the factors influencing adherence in children remain an important research focus. This study examines the sociodemographic, maternal, perinatal, and lifestyle determinants associated with MD adherence among children aged 6–9 years in an effort to identify key predictors and their impact on long-term nutritional habits. **Methods:** This study recruited 4851 children from diverse Greek rural and urban regions. The mothers of the enrolled children completed relevant questionnaires on their children’s sociodemographics, perinatal outcomes, anthropometric parameters, breastfeeding practices, and physical activity status. The enrolled children completed the Children’s Depression Inventory (CDI) and the State–Trait Anxiety Inventory for Children—State (STAIC-S) form to assess the presence of depression and anxiety symptoms, respectively. The KIDMED score was used to assess the MD compliance of the enrolled children. **Results:** The outcomes demonstrated that higher maternal education and family economic status gravitated toward increased MD adherence (*p* = 0.0071, *p* ˂ 0.0001), while exclusive breastfeeding (*p* ˂ 0.0001) and higher physical activity levels (*p* = 0.0101) were strong predictive factors for MD adherence, highlighting the role of early-life interventions in shaping dietary habits. In contrast, cesarean delivery (*p* = 0.0173) and higher birth weight (*p* ˂ 0.0001) were linked to lower MD adherence, indicating potential metabolic and behavioral predispositions. Notably, higher MD adherence correlated with lower prevalence of depressive and anxiety symptoms (*p* ˂ 0.0001, *p* = 0.0001), underscoring its potential protective role in mental health. **Conclusions:** Our findings highlight a complex interplay between early-life and dietary behaviors, while further longitudinal studies are needed to establish causality and optimize evidence-based nutritional strategies and education for childhood health and well-being.

## 1. Introduction

The Mediterranean diet (MD) has long been recognized as one of the most beneficial dietary patterns, promoting overall well-being through its balanced composition of fruits, vegetables, whole grains, fish, olive oil, and nuts. As a pattern rich in anti-inflammatory and antioxidant compounds, which also integrates physical activity, the MD has been linked to lower risks of numerous conditions, such as non-communicable diseases, cardiovascular disease, and diabetes, as well as neurodegenerative disorders [1,2,3,4]. Also, recent reports highlight that higher MD adherence may also have beneficial effects in the prevention and management of various endocrine disorders, improvement of bone health in older adults, particularly related to the mitigation of weight loss and bone mineral density decline, and improvement of male and female reproductive health [5,6]. Beyond its physical health advantages, recent studies also explore its potential role in mental health, evaluating how dietary habits may influence cognitive function and psychological resilience [7]. As such, the current literature suggests that adherence to the MD is associated with reduced symptoms of depression and anxiety, primarily due to its various nutraceuticals that support brain function [8]. Well-documented characteristics of compounds such as polyphenols found in olive oil, flavonoids from fruits and vegetables, and omega-3 from seafoods and nuts have been linked to the regulation of mechanisms critical for emotional stability, as well as lower prevalence rates of mood disorders and cognitive decline [9,10,11]. For instance, recent research highlights the gut–brain axis as a key pathway through which dietary components influence mood and cognitive function. In this setting, the MD, rich in prebiotics and polyphenols, supports gut microbiota diversity, which in turn regulates neurotransmitter production and stress response mechanisms [12,13,14].

While a vast variety of literature supports the psychological benefits of the MD in adults, several studies have also explored its impact on children and adolescents, given that early-life nutrition sets the stage for long-term psychological and physiological health [15,16]. In this setting, previous data have demonstrated that higher compliance with the MD is positively associated with better body weight management, reduced inflammation, and enhanced neurodevelopmental outcomes in children, highlighting how nutrition influences cognitive development, emotional regulation, and even susceptibility to mental health disorders later in life [7,17]. Also, a recent meta-analysis regarding adherence to the MD in children and adolescents has highlighted that MD-based interventions, with a mean intervention of 17 weeks, can be beneficial for the improvement of biomarkers of cardiometabolic health, such as total cholesterol, LDL cholesterol, HDL cholesterol, systolic blood pressure, and total glucose [18]. Additionally, adherence to the MD has also been linked to a lower risk of mood disorders and behavioral difficulties in childhood, suggesting that dietary choices could serve as a potential protective factor against psychological distress [19]. Ongoing research in this field has a key role in establishing such connections early so that future intervention may help attenuate the long-term psychological burden associated with poor nutrition.

In recent years, childhood anxiety and depression have emerged as significant public health concerns, with prevalence rates increasing worldwide. Given the developmental significance of diet, further investigations have focused on causal relationships between nutritional habits and psychological outcomes, examining this interplay within socioeconomic and environmental contexts. Beyond the direct effects of diet on health and well-being, socioeconomic status (SES) and parental dietary behaviors seem to play a significant role in shaping children’s nutritional habits and, consequently, their mental health. As current research highlights, families with lower SES often face barriers to accessing nutrient-rich foods, leading to poorer dietary quality and increased vulnerability to obesity, metabolic disorders, and psychological distress [20,21]. Additionally, previous data show that children from lower-income households are more likely to consume ultra-processed and calorie-dense foods, which are associated with higher risks of anxiety and depression [22].

Another key remark related to SES is how food accessibility and affordability shape children’s ability to maintain MD adherence. In this setting, recent trends in urbanization and the rising prevalence of ultra-processed foods have contributed to a shift away from healthy eating habits, potentially influencing both physical and mental health [23,24]. In Greece, recent findings have highlighted notable disparities in diet quality among children, driven by socioeconomic inequalities and variations in parental education [25]. As such, parental influence extends beyond economic factors, as dietary behaviors, mealtime practices, and education significantly impact children’s food choices. According to the 2019 pilot questionnaire distributed by the Greek Statistical Authority to monitor overall health levels in children aged 2 to 14 years, the data from 8125 respondents demonstrated that “moderate” and “bad/very bad” health represented 1.8% and 0.5% of the sample, respectively. Also, 6.7% of children aged 2 to 14 years, according to their parents/guardians, have a chronic problem or condition (a condition that is expected to last or last for more than 6 months with or without medication), and 2.2% have limited their outdoor or other activities due to health conditions, for at least 6 months [26]. Additionally, for the same year, most underweight (23.8%) children were recorded at ages 2 to 4 years, while most overweight or obese (43.0%) were at ages 5 to 7 years. Regarding the mothers’ health, 2019 data from the World Health Organization report showed a 20.3% prevalence of anemia in pregnant women aged 15–49 years [27]. Evidence suggests that children whose parents adhere to the MD are more likely to adopt healthier eating patterns, reinforcing the idea that household nutrition can play a crucial role in long-term physical and mental health outcomes [28]. Maternal dietary habits, particularly during pregnancy and early childhood, have been linked to neurodevelopment and emotional regulation, emphasizing the importance of early nutritional interventions [29]. In this setting, a previous study conducted in the U.S., involving 7798 participants, has demonstrated that higher adherence to the MD was associated with lower risks of adverse pregnancy outcomes, such as preeclampsia or eclampsia, gestational hypertension, gestational diabetes, preterm birth, etc. [30]. Also, a recent study conducted in Spain, involving 392 pregnant individuals assigned to the Mediterranean diet (from a total of 1221 participants), has demonstrated that adherence to a structured MD pattern was able to significantly reduce the percentage of newborns with birth weight below the 10th percentile [31]. While MD adherence remains a cultural staple, urbanization, economic fluctuations, and shifts in dietary accessibility have influenced children’s eating patterns and their potential mental health outcomes.

Understanding these socioeconomic and parental determinants is essential for developing effective interventions to promote healthy nutrition and psychological resilience in young populations. In this setting, this cross-sectional study aimed to (a) document the dietary habits and physical activity of children aged 6–9 years old and evaluate their correlation and (b) investigate how perinatal and socioeconomic factors of the mothers may contribute as predictors or protective factors to the risk of anxiety and depression in children aged 6–9 years old.

## 2. Materials and Methods

### 2.1. Study Population and Ethics

This cross-sectional survey included 5487 children aged 6–9 years and their mothers, recruited from ten regions across Greece (Athens, Thessaloniki, Larissa, Patra, Alexandroupolis, Kalamata, Ioannina, Crete, and South and North Aegean) between May 2018 and September 2022. Enrollment was randomized, with most participants assigned via primary education schools. Namely, via the personal conduct of the researchers with public schools of primary education, the study objectives were introduced to the mothers when visiting the schools. The mothers and children who met the inclusion criteria and were willing to volunteer for this study were enrolled.

**Figure 1:**
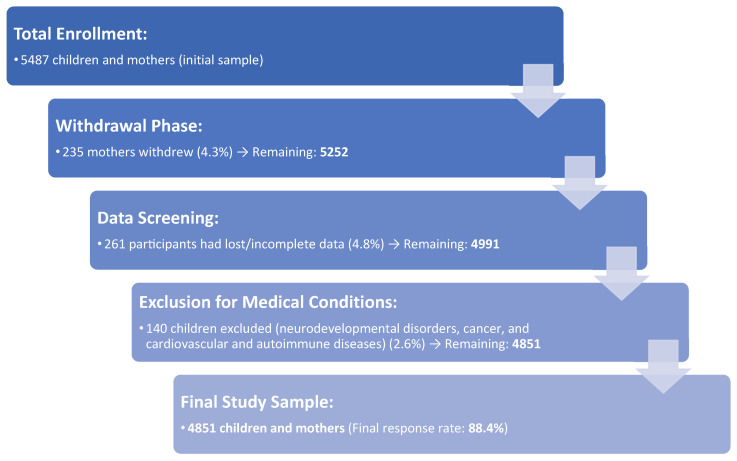
Enrollment flow chart.

*Inclusion criteria*: Children aged 6–9 without chronic diseases. *Exclusion criteria*: Neurodevelopmental disorders, cancer, and cardiovascular and autoimmune diseases (self-reported by mothers). Following withdrawals and exclusion due to incomplete data, the final study sample consisted of 4851 children and their mothers, resulting in a response rate of 88.4%. Ethical approval was granted by the Ethics Committee of the University of the Aegean (protocol no 12/14.5.2016), adhering to the WHO guidelines (52nd WMA General Assembly, Edinburgh, Scotland, 2000). Written informed consent was obtained, ensuring confidentiality. A sequence made the randomization of random binary numbers (i.e., 001, 110, and 110 in which 0 signified assignment and 1 non-assignment to the survey). Sample size calculations (PS software, software version 3.1.2) demonstrated a study power of 87.8%. A flow chart diagram of the study enrollment is depicted in Figure 1.

### 2.2. Study Design

#### 2.2.1. Sociodemographic Parameters

During the survey, relevant questionnaires were utilized to record the sociodemographic characteristics of the enrolled children and mothers via face-to-face interviews by trained nutritionists or dietitians to minimize recall bias. Mothers’ sociodemographic parameters, such as marital and employment status, smoking habits, and parity, were also collected. The maternal education level was classified into (a) primary education (<7 years of studies), (b) secondary education (7–12 years of studies), and (c) university studies (>12 years of studies). Financial level was categorized based on the annual family income in the following categories: <EUR 5000; EUR 5000–10,000; EUR 10,000–15,000; EUR 15,000–20,000; EUR 20,000–25,000; and >EUR 25,000, and additionally classified as low (≤EUR 10,000), medium (˃EUR 10,000 and ≤EUR 20,000), and high (˃EUR 20,000). This dual classification approach allows the analysis of financial status, enabling the identification of potential socioeconomic disparities in diet and health outcomes among children.

#### 2.2.2. Perinatal Outcomes and Breastfeeding Practices

Data on gestational weight gain (GWG), childbirth weight, and mode of delivery (vaginal or cesarean) were retrieved from the mothers’ medical records. Maternal BMI pre-pregnancy was measured by healthcare providers during early gestation visits, ensuring accuracy. The classification used for perinatal parameters for mothers and children is presented in Table 1, based on the Institute of Medicine (IOM) guidelines and the relevant literature [32,33,34,35]. Mothers’ and children’s body weights were determined by a Seca scale [Seca, Hanover, MD, USA], and height was determined by a portable stadiometer (GIMA Stadiometer 27335, Utrecht, The Netherlands). To evaluate pre-pregnancy maternal BMI, mothers’ anthropometric data during the first weeks of gestation were measured (not self-reported) during a visit to their gynecologists or public/private hospitals.

Breastfeeding practices were self-reported by the mothers, and exclusive breastfeeding for ≥4 months was used as a defined reference point for dietary transitions (gradually introducing pulp foods to the feeding practices at the end of the 4th month and the beginning of the 5th month) to minimize potential recall bias [29,36].

#### 2.2.3. Children’s Anthropometric Parameters and Physical Activity Evaluations

Children’s anthropometry parameters, such as body weight and height, were measured during the period of the survey by qualified staff. Body weight was measured using the same electronic scale, and height was assessed by a portable stadiometer [37,38,39]. Body weight was determined to the closest 100 g, and height was measured to the closest 0.50 cm. The International Obesity Task Force (IOTF) recommendations were applied for grouping the enrolled children as normal weight, overweight, or obese [40,41]. Physical activity levels were assessed using the International Physical Activity Questionnaire (IPAQ) long form, which categorizes activity levels as low, moderate, or high, based on the total duration of physical activity performed over the previous seven days. While IPAQ is a widely validated self-report tool, its application in younger populations presents potential limitations in accurately capturing activity levels. Therefore, its use in this study was intended primarily for stratification purposes rather than as a direct marker of physical activity intensity [42,43]. Moreover, mothers completed this questionnaire via face-to-face interviews by trained nutritionists or dietitians to minimize recall bias.

#### 2.2.4. Children’s Depression and Anxiety Evaluations

Depressive symptoms were evaluated using the Children’s Depression Inventory (CDI), a widely used self-report tool written at a first-grade reading level, containing 27 items. The CDI can be completed within 5–25 min by a child with age-appropriate reading abilities, and has well-established psychometric properties, ensuring accurate and reliable measurement of depression in children [44,45]. Additionally, anxiety symptoms were assessed via the State–Trait Anxiety Inventory for Children—State (STAIC-S) form, a 20-item self-report tool developed by Spielberger. The STAIC-S is one of the most frequently used instruments for evaluating childhood anxiety, demonstrating high reliability and satisfactory validity [46,47]. To ensure consistency in responses, qualified dietitians and nutritionists provided detailed, informative guidance to mothers and children regarding questionnaire completion and an analytical presentation of the questions. Mothers completed all study questionnaires except the CDI and STAIC-S, which were self-reported by children.

#### 2.2.5. Evaluations of Children’s MD Adherence

Children’s MD adherence was assessed using the KIDMED questionnaire, one of the most extensively applied scoring systems for evaluating diet quality [48]. This 16-item tool includes 12 positively associated items (e.g., consumption of fruits, vegetables, whole grains, fish, and olive oil) and 4 negatively associated items (e.g., frequent intake of fast food, sweets, and skipping breakfast). The scores range from 0 to 12, categorized as ≥8 points: high adherence, 4–7 points: moderate adherence, and ≤3 points: low adherence. A summary of all evaluations used in the current study is presented in Table 2.

### 2.3. Statistical Analysis

Continuous variables were analyzed using Student’s *t*-test, with normal distribution verified via the Kolmogorov–Smirnov test. Chi-square tests were performed for categorical variables. The results were expressed as the mean ± standard deviation (SD) for quantitative variables and absolute or relative frequencies for qualitative variables. A multivariate binary logistic regression analysis assessed whether MD adherence was independently associated with sociodemographic and anthropometric factors, perinatal outcomes, and lifestyle characteristics, including childhood depression and anxiety, while adjusting for potential confounders. Statistical analyses were conducted using Statistica 10.0 software (Informer Technologies, Hamburg, Germany).

## 3. Results

An extended summary of the descriptive demographic, maternal, birth, and child health characteristics of the current study (n = 4851) is presented in Table 3. Our study focused on several key socioeconomic indicators, including maternal health factors, early childhood parameters, and lifestyle attributes relevant to the investigation of dietary influences on mental health outcomes. In this setting, our study population of children exhibited a balanced gender distribution (49.7% male, 50.3% female), with children participating in the survey being 7.4 ± 1.3 years old, and the majority of them are of Greek nationality (95.7%) and residing in urban areas (65.8%). Additionally, the children’s physical activity levels were largely low (47.8%), while the MD adherence evaluation also demonstrated that the majority of children fell into the low (39.7%) and moderate (37.6%) classes of KIDMED. Our outcomes also revealed considerable prevalence of depressive and anxiety symptoms (30.0% and 29.4%, respectively).

Regarding the outcomes of the mothers’ responses, it was noted that despite a relatively high maternal employment rate (68.8%), a significant portion of families (42.0%) fell into low-income categories, highlighting potential socioeconomic inequalities. This was further evident in maternal education, where nearly a third of mothers had a low educational level (30.5%), which relevant research highlights as an important factor in shaping dietary habits. Regarding maternal health, overweight and obesity were present in 21.6% of mothers before pregnancy, with gestational weight gain exceeding IOM recommended thresholds in 38.8% of cases. It is worth noting that the mode of delivery was mostly cesarean section (56.0%), while exclusive breastfeeding was reported in 50.1% of children.

In the analysis of cross-tabulation, the potential associations between the children’s MD compliance with the collected variables were examined (Table 4). In this setting, the results revealed that increased adherence to the MD was essentially more frequent in boys than girls (*p* = 0.0223), while high MD adherence was positively correlated with higher education levels of the mothers and family financial status (*p* = 0.0071 and *p* ˂ 0.0001, respectively). Also, children whose mothers were unemployed exhibited higher MD adherence than those whose mothers were employed (*p* = 0.0385), and children from married households showed lower MD compliance compared to those from divorced households (*p* = 0.0002), underscoring a potential complexity in the influence of household dynamics over dietary and food choices. Additionally, maternal pre-pregnancy overweight and obesity were significantly more frequent among children with lower MD adherence (*p* = 0.0339), indicating a potential early-life nutritional link, while higher childbirth weight was associated with lower MD adherence (*p* ˂ 0.0001). The delivery method also played a role, as children born via cesarean section exhibited lower MD adherence compared to those born vaginally (*p* = 0.0173).

Finally, exclusive breastfeeding emerged as a strong predictor of higher MD adherence (*p* ˂ 0.0001), reinforcing its potential long-term benefits on dietary habits, while low physical activity was significantly associated with low MD adherence levels compared to children with moderate or high physical activity (*p* ˂ 0.0001).

**Figure 2:**
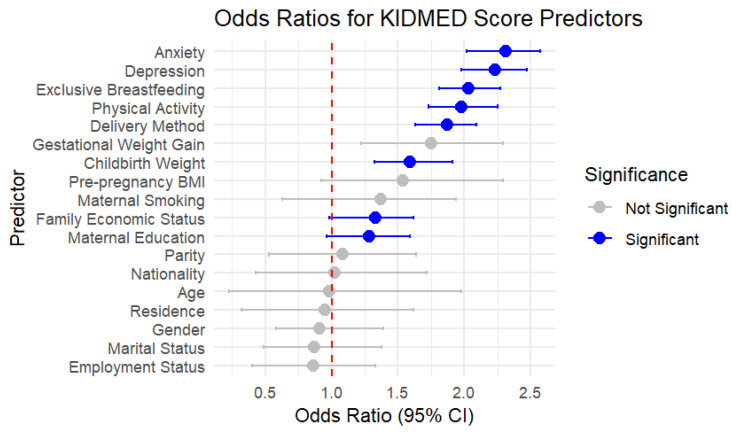
Multivariate logistic regression analysis for children’s MD adherence (low vs. moderate/high).

In the binary logistic regression analysis, the educational level of the mothers, family economic status, childbirth weight, method of delivery, exclusive breastfeeding (over the first four months), children’s physical activity, depression, and anxiety were significantly and independently associated with children’s MD adherence (Table 5, Figure 2). In fact, higher adherence to the MD trended toward an association with higher education levels of the mothers (OR = 1.28, 95% CI: 0.96–1.59, *p* = 0.0292) and family financial status (OR = 1.33, 95% CI: 0.98–1.62, *p* = 0.0093), though the confidence intervals suggest these effects may not be definitive. Additionally, children with higher birth weight were significantly more likely to have lower MD adherence (OR = 1.59, 95% CI: 1.32–1.91, *p* = 0.0109), underscoring the potential metabolic influences of early dietary patterns, while similar observations were relevant regarding the delivery method, as children born via cesarean section had lower MD adherence compared to those born vaginally (OR = 1.87, 95% CI: 1.63–2.09, *p* = 0.0323), noting that children born by cesarean section showed an 87% higher likelihood of presenting low MD compliance. In contrast, maternal pre-pregnancy BMI status and gestational weight gain did not show statistically significant effects (*p* = 0.2422 and *p* = 0.2903, respectively).

It is worth noting that exclusive breastfeeding emerged as a strong predictor of higher MD adherence (OR = 2.03, 95% CI: 1.81–2.27, *p* = 0.0098), underlining the long-term influences of early feeding practices. Likewise, children with higher physical activity levels demonstrated significantly higher adherence to the MD (OR = 1.98, 95% CI: 1.73–2.25, *p* = 0.0101), underscoring the behavioral connections between active lifestyles and dietary choices that the literature has highlighted as relevant for adults as well. In both cases, children not receiving exclusive breastfeeding, as well as those with low physical activity, had a two-fold higher probability of presenting low MD adherence levels. Notably, low adherence to MD was significantly associated with increased prevalence of depressive and anxiety symptoms, indicating a more than two-fold higher risk of developing depressive and anxiety symptoms, with depression (OR = 2.23, 95% CI: 1.98–2.47, *p* = 0.0036) and anxiety (OR = 2.31, 95% CI: 2.02–2.57, *p* = 0.0067) showing strong statistical significance.

## 4. Discussion

Nutrition plays a critical role in human development and the management of health throughout the lifespan. Several elements of our diet are directly linked to the prevention of various diseases, while ongoing research keeps highlighting the damaging effects that poor nutrition can have [49,50]. As such, the dietary habits established through the early stages of life have a pivotal role in the direction of our physical and mental health later in life [51]. This study has demonstrated crucial insights into the determinants of MD adherence among children, consolidating the significant influence of socioeconomic, maternal, and lifestyle factors.

Notably, socioeconomic factors such as higher maternal education and economic status exhibited a trend toward promoting better MD adherence, highlighting the interplay between education, economic stability, and nutritional habits being developed from the early stages of life. The positive association between higher maternal education and MD adherence aligns with previous findings indicating that maternal knowledge and awareness of nutrition play a crucial role in shaping children’s dietary habits [52,53,54]. Educated mothers are more likely to prioritize balanced nutrition, understand the benefits of the MD, and implement healthier food choices at home. Such findings might also be relevant to a much-needed transition toward healthier and more sustainable diets [55]. In this setting, the economic cofounder is also highly relevant to food choices and dietary patterns. Our findings have demonstrated that higher family economic status has been linked to greater adherence to the MD, indicating better access to diverse and nutrient-rich foods, and thus reducing reliance on processed or low-quality diets. However, these associations have been marked as borderline significant, which is a potential indicator of the trends in affordability of healthy food and a global concern regarding food insecurity [56,57,58].

Interestingly, maternal employment status showed an inverse association, with children of unemployed mothers exhibiting higher MD adherence. This may reflect a greater perspective regarding the impact of household dynamics of a stay-at-home parent in their involvement in meal preparation and dietary supervision, and in limiting time constraints that lead to convenience-based food choices [59,60]. In contrast, marital status did not show a strong association, which is consistent with prior studies suggesting that household structure alone does not significantly impact healthy dietary patterns when socioeconomic factors are accounted for [61].

Additionally, maternal pre-pregnancy overweight and obesity were significantly more frequent among children with lower MD adherence, indicating a potential early-life nutritional link, while higher childbirth weight was associated with lower MD adherence, pointing to possible metabolic predispositions affecting dietary choices later in childhood. Such findings allow us to highlight plausible correlations between the role of early-life nutrition and later outcomes; however, causality could be established in the context of a longitudinal cohort study. Also, exclusive breastfeeding emerged as a strong predictor of higher MD adherence, reinforcing its long-term benefits on dietary habits. Breastfed children tend to develop healthier taste preferences, particularly for natural and minimally processed foods, which align with MD principles [62,63].

Furthermore, the finding that higher birth weight was associated with lower MD adherence is noteworthy, as birth weight has been linked to early metabolic programming that may influence food preferences and energy balance later in life [64,65]. Unexpectedly, children born via cesarean section and those with higher birth weight displayed lower MD adherence, suggesting potential metabolic or early-life influences on dietary habits. Similarly, children born via cesarean section exhibited lower MD adherence, which may be related to gut microbiota differences compared to vaginally delivered infants [66,67,68]. As previous research suggests, cesarean-born children may have altered microbiome compositions, potentially affecting metabolism and dietary behaviors, while this also indicates a link to breastfeeding practices.

Additionally, although higher physical activity levels were significantly associated with greater MD adherence, supporting the well-established link between active lifestyles and healthier eating behaviors, the overall low levels of physical activity reported in children suggest an emerging concern about the development of exercise behaviors that can impact their health later in life.

Moreover, the observed association between MD adherence and lower prevalence of depressive and anxiety symptoms underscores the potential mental health benefits of diet and is consistent with growing evidence supporting the protective effects of the MD on mental health, though further investigation is warranted to explore causality. The MD is rich in omega-3 fatty acids, polyphenols, and essential micronutrients, all of which contribute to neuroprotection and mood regulation. In this aspect, a recent meta-analysis study revealed an inverse significant association between MD adherence and the risk of depression when separately examining four prospective studies and eight cross-sectional studies [69]. However, the currently available clinical studies were performed in young, middle-aged, and older adults or adolescents, but not in children [69,70,71,72]. On the other hand, the potential association of MD adherence with anxiety risk is generally less studied, being restricted to adult populations. In this respect, there are mainly cross-sectional studies that revealed a significant inverse association between MD adherence and the risk of anxiety [73,74,75,76]. Namely, a recent 2023 study conducted in Chile, involving 934 university students, demonstrated that moderate and high adherence to the Mediterranean diet showed lower odds of depression, as evaluated by the Depression Anxiety and Stress Scale (DASS-21 > 5) [73]. In a similar setting, a 2024 study in Türkiye, also involving university students (N = 750), reported that lower MD adherence, as evaluated via the KIDMED score, had a negative impact on the Depression Anxiety and Stress Scale (DASS-42) for male and female students [74]. Investigating the adult population, particularly those struggling with obesity, a recent cross-sectional study involving 4957 patients demonstrated that each 1-point increase in the 14-item MEDAS score resulted in a decrease of 0.18 points (95%CI: −0.33, −0.03) and 0.10 points (95%CI: −0.16, −0.03) in the STAI2 (20-item STAI2 questionnaire) and QD (24-item QD questionnaire) scores, respectively [75]. These findings further underline the direct link between MD adherence and psychological distress, while also demonstrating the complex relations of confounding factors in adult populations.

### Study Limitations and Considerations

While this study suggests a beneficial relationship between dietary habits and psychological distress, reverse causality cannot be ruled out, as children experiencing psychological distress may also be more conscious of dietary health or receive greater parental intervention in nutrition. In addition, while this study provides valuable insights, the cross-sectional nature of the analysis prevents causal inference, and potential confounding factors such as parental dietary habits and food availability were not directly assessed. It is worth noting that the investigation of MD adherence in the setting of a country such as Greece, which is considered culturally relevant to its context, is expected to differ from studies conducted in countries or settings with other cultural dietary traditions, accessibility to Mediterranean foods, and societal attitudes toward nutrition. As such, these factors could in turn impact adherence in children from different backgrounds.

Also, in the setting of clinical practice and research, future investigations should explore longitudinal associations and incorporate biomarkers to better understand the physiological mechanisms linking MD adherence to mental health outcomes, such as depression and anxiety, while the role of the mothers’ gynecologists regarding dietary consultation and potential referral to a registered dietitian at any stage of the pregnancy is a key item that calls for further investigations, as it brings together maternal health, dietary guidance, and psychological well-being. A more integrated approach to maternal nutrition, incorporating both medical and dietary expertise, could offer significant benefits in shaping mental health outcomes from early childhood. Therefore, further interdisciplinary research and clinical practices should aim to refine these connections, ensuring comprehensive support for both mothers and children that could very well lead to healthier adolescence.

## 5. Conclusions

Overall, this work highlights the complex interplay between socioeconomic, maternal, perinatal, and lifestyle factors in shaping children’s dietary behaviors. The findings reinforce the importance of creating an effective framework of nutrition education that includes early-life interventions implemented by a multidisciplinary team of experts, such as dietitians, pediatricians, gynecologists, and psychologists, promoting MD adherence and physically active lifestyles. Given the potential mental health benefits observed, further research is warranted to explore causal pathways and optimize dietary recommendations for childhood well-being. Interventional studies assessing the potential beneficial effects of the MD against the severity of childhood depression and anxiety symptoms are highly recommended. Public health strategies and policies should be applied by educating future mothers about the healthy dietary patterns and lifestyle factors that can act as preventive agents against childhood depression and anxiety, while taking into account the socioeconomic disparities of the population.

## Figures and Tables

**Table 1:** Classification of perinatal and child parameters.

Parameter	Categories	Ref.
**Gestational Weight Gain (GWG)**	Underweight: 12.5–18 kg Normal weight: 11.6–16 kg Overweight: 7–11.5 kg Obese: 5–9 kg	[32,33]
**Childbirth Weight**	Low: <2500 g Normal: 2500–4000 g High: >4000 g	[34,35]

**Table 2:** Overview of study evaluations.

Category	Assessment Method	Key Variables	Measurement Tools
**Sociodemographic Parameters**	Face-to-face interviews with mothers	Child age, gender, nationality, and residence; maternal education, income, marital/employment status, smoking habits, and parity	Standardized sociodemographic survey [37,38,39]
**Perinatal Outcomes**	Medical records	Gestational weight gain, childbirth weight, and mode of delivery	IOM guidelines for GWG, birth weight classification [32,33,34,35]
**Anthropometric Parameters**	Direct measurements by trained staff	Body weight, height	Seca scale, GIMA stadiometer, and IOTF classification [40,41]
**Physical Activity**	Self-reported questionnaire	Weekly activity duration	IPAQ survey [42,43]
**Depression and Anxiety**	Child self-report	Depressive symptoms, anxiety levels	CDI and STAIC-S [44,45,46,47]
**Mediterranean Diet Adherence**	Child self-report	Dietary behaviors	KIDMED questionnaire [48]

**Table 3:** Descriptive statistics of the study population.

Characteristics (n = 4851)		Descriptives, n (%)
**Children**		
**Age (mean ± SD; years)**		7.4 ± 1.3
**Gender**	Male	2413 (49.7)
	Female	2430 (50.3)
**Nationality**	Greek	4643 (95.7)
	Other	203 (4.3)
**Type of residence**	Urban	3190 (65.8)
	Rural	1661 (34.2)
**Childbirth weight**	Low (<2500 g)	407 (8.4)
	Normal (2500–4000 g)	4171 (86.0)
	High (>4000 g)	273 (5.6)
**Physical activity**	Low	2320 (47.8)
	Moderate	1873 (38.6)
	High	658 (13.6)
**Children depression**	No	3394 (70.0)
	Yes	1457 (30.0)
**Children anxiety**	No	3426 (70.6)
	Yes	1425 (29.4)
**KIDMED score**	Low	1927 (39.7)
	Moderate	1823 (37.6)
	High	1101 (22.7)
**Mothers’**		
**Educational level**	Low	1481 (30.5)
	Moderate	2079 (42.9)
	High	1291 (26.6)
**Family economic status**	Low	2037 (42.0)
	Moderate	1840 (37.9)
	High	974 (20.1)
**Smoking habits**	No smokers	3602 (74.2)
	Regular smokers	1249 (25.8)
**Employment status**	Employed	3192 (68.8)
	Unemployed	1659 (34.2)
**Marital status**	Married	3521 (72.6)
	Divorced	1330 (27.4)
**Parity**	Nulliparity	3262 (67.2)
	Multiparity	1589 (32.8)
**Pre-pregnancy BMI status**	Underweight	144 (3.0)
	Normal weight	3661 (75.5)
	Overweight	829 (17.1)
	Obese	217 (4.5)
**Gestational weight gain**	Low	713 (14.7)
	Normal	2256 (46.5)
	Excessive	1882 (38.8)
**Kind of delivery**	Vaginal	2136 (44.0)
	Caesarean section	2715 (56.0)
**Exclusive breastfeeding**	No	2421 (49.9)
	Yes	2430 (50.1)

**Table 4:** Associations of KIDMED score with relevant study evaluations.

Characteristics (n = 4851)	KIDMED Score, n (%)			
	Low 1927 (39.7)	Moderate 1823 (37.6)	High 1101 (22.7)	*p*-Value
**Gender**				0.0223
*Male*	916 (47.5)	918 (50.4)	579 (52.6)	
*Female*	1011 (52.5)	905 (49.6)	522 (47.4)	
**Educational level**				0.0071
*Low*	624 (32.4)	514 (28.2)	343 (31.1)	
*Moderate*	826 (42.9)	813 (44.6)	440 (40.0)	
*High*	477 (24.7)	496 (27.2)	318 (28.9)	
**Economic status**				˂0.0001
*Low*	822 (42.7)	776 (42.6)	439 (39.9)	
*Moderate*	775 (40.2)	682 (37.4)	383 (34.8)	
*High*	330 (17.1)	365 (20.0)	279 (25.3)	
**Employment**				0.0385
*Employed*	1301 (67.5)	1196 (65.6)	695 (63.1)	
*Unemployed*	626 (32.5)	627 (34.4)	406 (36.9)	
**Marital status**				0.0002
*Married*	1427 (74.1)	1349 (74.0)	745 (67.7)	
*Divorced*	500 (25.9)	474 (26.0)	356 (32.3)	
**Parity**				0.0114
*Nulliparity*	1254 (65.1)	1234 (67.7)	774 (70.3)	
*Multiparity*	673 (34.9)	589 (32.3)	327 (29.7)	
**Pre-pregnancy BMI**				0.0339
*Underweight*	59 (3.1)	49 (2.7)	36 (3.4)	
*Normal weight*	1436 (74.5)	1386 (76.0)	839 (76.2)	
*Overweight*	325 (16.9)	327 (17.9)	177 (16.8)	
*Obese*	107 (5.5)	61 (3.4)	49 (4.6)	
**Gestational weight gain**				0.1375
*Low*	278 (14.4)	295 (16.2)	140 (12.7)	
*Normal*	903 (46.9)	825 (45.3)	528 (48.0)	
*Excessive*	746 (38.7)	703 (38.5)	433 (39.3)	
**Childbirth weight**				˂0.0001
*Low*	124 (6.4)	148 (8.1)	110 (10.0)	
*Normal*	1533 (79.6)	1535 (84.2)	919 (83.5)	
*High*	270 (14.0)	140 (7.7)	72 (6.5)	
**Kind of delivery**				0.0173
*Vaginal*	802 (41.6)	822 (45.1)	512 (46.5)	
*Caesarean section*	1125 (58.4)	1001 (54.9)	589 (53.5)	
**Exclusive breastfeeding**				˂0.0001
*No*	1048 (54.4)	874 (47.9)	499 (45.3)	
*Yes*	879 (45.6)	949 (52.1)	602 (54.7)	
**Physical activity**				˂0.0001
*Low*	985 (51.1)	885 (48.6)	450 (40.9)	
*Moderate*	735 (38.2)	695 (38.1)	443 (40.2)	
* High *	207 (10.7)	243 (13.3)	208 (18.9)	
**Depression**				˂0.0001
*No*	1268 (65.8)	1320 (72.4)	806 (73.2)	
*Yes*	659 (34.2)	503 (27.6)	295 (26.8)	
**Anxiety**				0.0001
*No*	1295 (67.2)	1314 (72.1)	817 (74.2)	
*Yes*	632 (32.8)	509 (27.9)	284 (25.1)	

**Table 5:** Multivariate logistic regression analysis for children’s MD adherence.

Characteristics	KIDMED Score (Low vs/Moderate/High)	
	OR * (95% CI **)	*p*-Value
**Age** (Over/Below mean value)	0.98 (0.23–1.98)	0.7898
**Gender** (Male/Female)	0.91 (0.58–1.39)	0.1282
**Nationality** (Greek/Other)	1.02 (0.43–1.72)	0.4503
**Type of residence** (Rural/Urban)	0.95 (0.32–1.62)	0.7346
**Maternal educational level** (Low/Moderate and High)	1.28 (0.96–1.59)	**0.0292**
**Family economic status** (Low/Moderate and High)	1.33 (0.98–1.62)	**0.0093**
**Maternal smoking status** (Regular smokers/Non-smokers)	1.37 (0.63–1.94)	0.2892
**Employment status** (Unemployed/Employed)	0.86 (0.40–1.33)	0.4364
**Marital status** (Married/Divorced)	0.87 (0.49–1.38)	0.2010
**Parity** (Multiparity/Nulliparity)	1.08 (0.53–1.64)	0.3292
**Maternal pre-pregnancy BMI status** (Overweight and obesity/Underweight and normal weight)	1.54 (0.92–2.29)	0.2422
**Maternal gestational weight gain** (Low and Excessive/Normal)	1.75 (1.22–2.29)	0.2903
**Childbirth weight** (High/Normal and low)	1.59 (1.32–1.91)	**0.0109**
**Kind of delivery** (Caesarean section/Vaginal)	1.87 (1.63–2.09)	**0.0323**
**Exclusive breastfeeding** (No/Yes)	2.03 (1.81–2.27)	**0.0098**
**Physical activity** (Low/Moderate and High)	1.98 (1.73–2.25)	**0.0101**
**Depression** (Yes/No)	2.23 (1.98–2.47)	**0.0036**
**Anxiety** (Inadequate/Adequate)	2.31 (2.02–2.57)	**0.0067**

* OR: Odds Ration, and ** CI: Confidence Interval.

## Data Availability

All relevant data summarized in this study are available upon request from the principal investigator, Constantinos Giaginis.
